# Exosomes Released from Mycoplasma Infected Tumor Cells Activate Inhibitory B Cells

**DOI:** 10.1371/journal.pone.0036138

**Published:** 2012-04-27

**Authors:** Chenjie Yang, Geetha Chalasani, Yue-Harn Ng, Paul D. Robbins

**Affiliations:** 1 Department of Microbiology and Molecular Genetics, University of Pittsburgh School of Medicine, Pittsburgh, Pennsylvania, United States of America; 2 Renal-Electrolyte Division, Departments of Medicine and Immunology, University of Pittsburgh School of Medicine, Pittsburgh, Pennsylvania, United States of America; 3 Thomas E. Starzl Transplantation Institute, University of Pittsburgh School of Medicine, Pittsburgh, Pennsylvania, United States of America; Universidade de Sao Paulo, Brazil

## Abstract

Mycoplasmas cause numerous human diseases and are common opportunistic pathogens in cancer patients and immunocompromised individuals. Mycoplasma infection elicits various host immune responses. Here we demonstrate that mycoplasma-infected tumor cells release exosomes (myco+ exosomes) that specifically activate splenic B cells and induce splenocytes cytokine production. Induction of cytokines, including the proinflammatory IFN-γ and the anti-inflammatory IL-10, was largely dependent on the presence of B cells. B cells were the major IL-10 producers. In splenocytes from B cell deficient μMT mice, induction of IFN-γ+ T cells by myco+ exosomes was greatly increased compared with wild type splenocytes. In addition, anti-CD3-stimulated T cell proliferation was greatly inhibited in the presence of myco+ exosome-treated B cells. Also, anti-CD3-stimulated T cell signaling was impaired by myco+ exosome treatment. Proteomic analysis identified mycoplasma proteins in exosomes that potentially contribute to the effects. Our results demonstrate that mycoplasma-infected tumor cells release exosomes carrying mycoplasma components that preferentially activate B cells, which in turn, are able to inhibit T cell activity. These results suggest that mycoplasmas infecting tumor cells can exploit the exosome pathway to disseminate their own components and modulate the activity of immune cells, in particular, activate B cells with inhibitory activity.

## Introduction

Exosomes are 30–100 nm membrane vesicles released by a wide variety of cells. They are formed by reverse budding of the multivesicular bodies in the late endocytic compartments and are released upon their fusion with the plasma membrane [1], [2]. The protein composition of exosomes usually reflects that of the parental cells [3]. Exosomes have been shown to have various immunoregulatory effects, which also largely depend on the nature of the parental cells. Dendritic cell (DC)-derived exosomes can be either immunostimulatory or immunosuppressive, provided different inducing conditions [4], [5], [6]. Tumor-derived exosomes were initially considered as a new source of tumor antigens that could be used to stimulate anti-tumor responses [7]. However, tumor-derived exosomes have also been found to possess diverse immunosuppressive properties, such as negatively regulating the function of antigen-presenting cells (APCs) and effector cells (e.g. natural killer cells and T cells), promoting the generation of myeloid suppressor cells, and supporting the function of regulatory T cells [8], [9], [10], [11], [12], [13], [14]. Interestingly, studies have shown that intracellular pathogens infecting APCs can modulate the immunoregulatory properties of APC-derived exosomes, making them proinflammatory [15], [16] or mitogenic [17].

Mycoplasmas are parasitic bacteria of minute size (0.2–1.0 µm), causing numerous diseases such as pneumonia and also acting as opportunistic pathogens that colonize a host with a weak immune system [18], [19], [20]. They can infect many cell types by either surface attachment to the cell membrane or fusion with the host cells [20]. Persistent mycoplasma infection induces chromosomal instability and malignant transformation of mammalian cells [21], [22], [23], [24], [25], [26], [27], and certain tumor-associated proteins are proposed to have a mycoplasma origin [28]. Mycoplasma infection of tumor cells were reported to increase tumor cell invasiveness [29]. Mycoplasmas can induce a wide range of immune responses. Many mycoplasma species can activate monocytes and induce the secretion of proinflammatory cytokines [30], [31], [32]. Mycoplasmas can also induce immunosuppression through various mechanisms including arginine depletion, cytotoxicity and induction of anti-inflammatory cytokines [20], [30], [33], [34], [35]. In addition, temporary inhibition of cell-mediated or humoral immune responses by mycoplasma infection was observed in different hosts [36], [37], [38].

The incidence of mycoplasma infection in established tumors is unclear. Nevertheless, mycoplasma DNA has been detected in different archived human cancer tissues, including ovarian cancer, gastric carcinoma, colon carcinoma, esophageal cancer, lung cancer, breast cancer and glioma, suggesting the possible co-existence of mycoplasmas and tumors *in vivo*. [39], [40]. *In vitro*, mycoplasma infection is commonly found in laboratory cultured cell lines including tumor cell lines [41]. During the study of tumor-derived exosomes, we found that certain immune responses elicited by exosomes were associated with mycoplasma infection of the parental tumor cells. Here we report that exosomes derived from mycoplasma-infected tumor cells preferentially activate B cells and induce robust cytokine production by splenocytes, including both proinflammatory and anti-inflammatory cytokines. In addition, T cell activation and proliferation is inhibited by those activated B cells. Our data indicate that mycoplasmas are able to exploit the exosome pathway of the host tumor cells to disseminate their own components and influence the activity of immune cells. Our results also suggest a potential immunosuppressive mechanism of mycoplasmas-infected tumor cells through the release of exosomes.

## Results

### Exosomes derived from mycoplasma-infected tumor cells induce splenocytes cytokine production and splenic B cell activation

**Figure 1:**
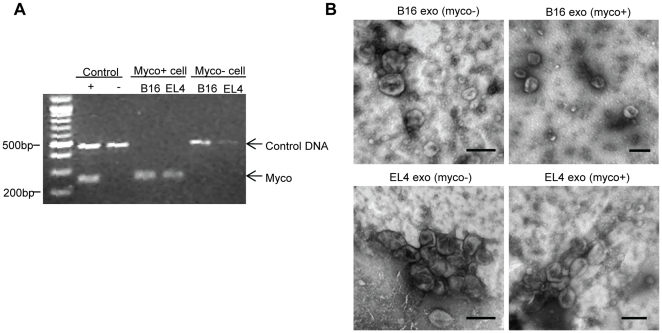
Morphology of exosomes derived from healthy or mycoplasma-infected tumor cells. (A) Detection of mycoplasma DNA in B16 and EL4 cell cultures. Culture supernatants were tested by PCR using primer sets specific to the highly conserved 16S rRNA coding region in the mycoplasma genome. Mycoplasma positive samples show bands in the range of 260±8 bp. An internal control DNA band at 481 bp was included and is attenuated in the presence of heavy mycoplasma DNA load. (B) Electron micrograph of exosomes prepared from non-infected (myco−) and infected (myco+) tumor cell cultures. Scale bar: 100 nm.

Tumor cell lines can be infected with mycoplasmas during long-term culture, with no apparent alterations on cell growth and proliferation. A screen for mycoplasma infection of a panel of murine tumor lines identified subcultures of the B16 melanoma cells and the EL4 thymoma cells as mycoplasma positive (Figure 1A). Exosomes were isolated from the culture supernatants of both infected and non-infected cell lines. Similar amounts of exosome proteins were obtained and electron microscopy (EM) demonstrated that exosomes derived from infected cells (myco+ exosomes) have similar morphology as those derived from healthy cells (myco− exosomes). No mycoplasma-like bacteria were observed by EM in myco+ exosome preparations (Figure 1B).

**Figure 2:**
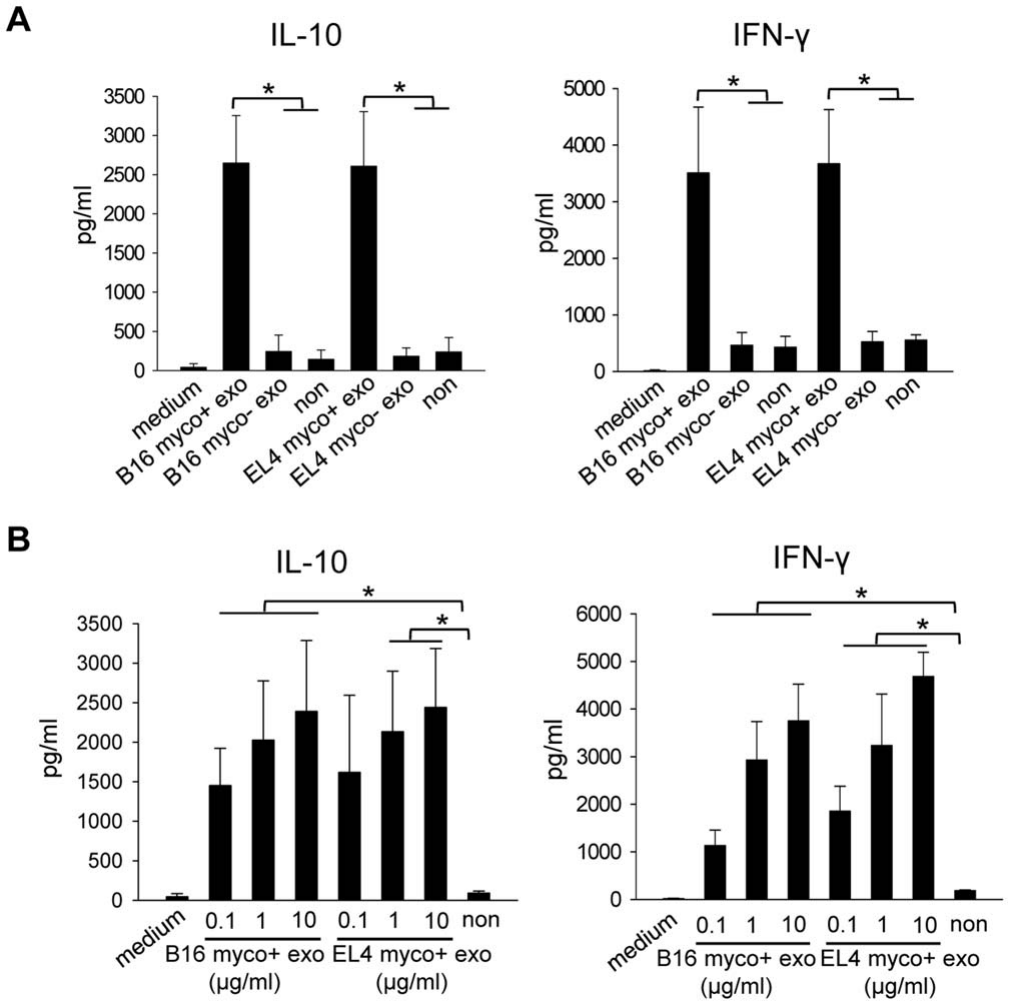
Cytokine induction in splenocytes by myco+ exosome treatment. (A) Splenocytes from C57BL/6 mice were cultured in a 24-well-plate at the density of 5×10^6^ cells/1.5 ml media/well in the presence of 30 U/ml rmIL-2 and were treated with either myco+ exosomes or myco− exosomes (1 µg/ml), or left untreated for 72 hr. The IL-10 and IFN-γ levels (pg/ml) in the culture supernatants were measured by ELISA. Treatments were conducted in duplicates or triplicates in each experiment. Data represent the averaged cytokine levels ± SD of three independent experiments. (B) Dose-dependent cytokine induction by myco+ exosomes. Splenocytes were treated with an increasing dose of myco+ exsosomes (0.1, 1 and 10 µg/ml) for 72 hr, and the cytokine levels were measured by ELISA. Treatments were conducted in duplicates. Data represent the averaged cytokine levels ± SD of three independent experiments. Significance at: *, P<0.05.

To examine the effect of myco+ exosomes on immune cells, splenocytes from C57BL/6 mice were treated with 1 µg/ml of exosomes for 72 hr. Treatment with myco+ exosomes resulted in robust induction of the proinflammatory cytokine IFN-γ as well as the anti-inflammatory cytokine IL-10, whereas myco− exosomes did not induce cytokine production (Figure 2A). The cytokine induction by myco+ exosomes was dose-dependent (Figure 2B). Similar results were obtained using either B16 or EL4 exosomes.

**Figure 3:**
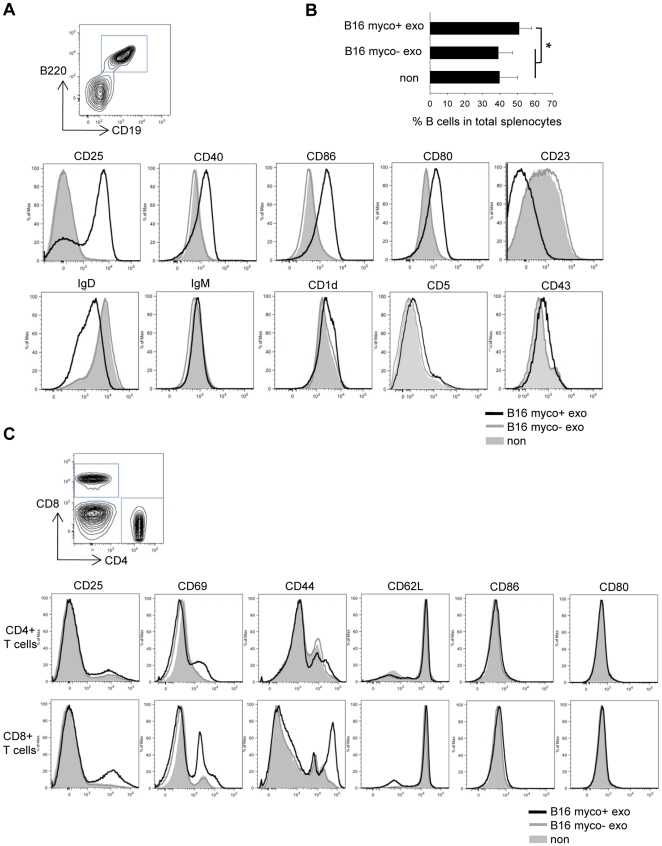
Myco+ exosomes induce B cell activation and expansion. Splenocytes were treated with 1 µg/ml of B16 myco+ exosomes or B16 myco− exosomes, or cultured untreated for 72 hr. Cells were harvested and analyzed by FACS. (A) Expression of CD25, CD40, CD86, CD80, CD23, IgD, IgM, CD1d, CD5 and CD43 in the B cell gate (CD19+B220+). (B) Percentage of B cells in total splenocytes within the live cell gate after exosome treatment. Data represents the mean ± SD of four independent experiments. Significance at: *, P<0.05. (C) Expression of CD25, CD69, CD44, CD62L, CD80 and CD86 in the CD4+ T cell gate and the CD8+ T cell gate. Black line: B16 myco+ exosome treatment; grey line: B16 myco− exosome treatment; grey solid: untreated.

Myco+ exosome treatment also resulted in splenic B cell activation, as evidenced by CD25^hi^, CD40^hi^, CD86^hi^, CD80^hi^ and IgD^lo^ expression on B220+CD19+ cells. There was no significant change in the expression of IgM, CD1d, and CD5 on B cells, suggesting that myco+ exosomes were not preferentially stimulating either marginal zone or B1 B cells expressing these markers. In contrast, myco− exosomes did not stimulate B cells (Figure 3A). Also, an increase in the percentage of B cells in total splenocytes was observed after myco+ exosome treatment (Figure 3B). Myco+ exosome treatment also resulted in moderate T cell activation, as evidenced by increased CD44^hi^, CD69^hi^, CD25^hi^, CD62L^lo^ CD8+ T cells and increased CD69^hi^ CD4+ T cells (Figure 3C). Similar results were obtained with either B16 or EL4 exosomes (data not shown).

### Cytokine induction by myco+ exosome is largely dependent on the presence of B cells

**Figure 4:**
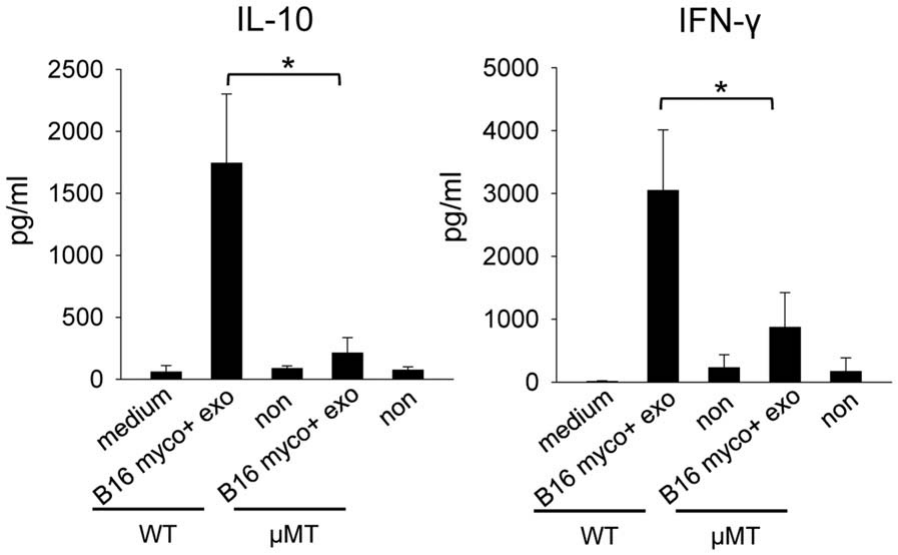
Cytokine induction by myco+ exosomes in WT and μMT splenocytes. Splenocytes from either WT mice or μMT mice were cultured in 24-well-plate at 5×10^6^ cells/1.5 ml media/well with 30 U/ml of rmIL-2 and treated with 1 µg/ml of B16 myco+ exosomes or left untreated for 72 hr. IL-10 and IFN-γ levels in the culture supernatants were measured by ELISA. Treatments were conducted in triplicates in each experiment. Data represents the averaged cytokine levels ± SD of three independent experiments. Significance at: *, P<0.05.

To determine if cytokine induction by myco+ exosome correlates with B cell activation, we examined the cytokine production of splenocytes isolated from B cell deficient μMT mice upon exosome treatment. Splenocytes isolated from wide type (WT) mice or μMT mice were treated with 1 µg/ml of B16 myco+ exosomes for 72 hr and the levels of IL-10 and IFN-γ in the culture supernatants were tested. Interestingly, there was a significant reduction in the amount of both cytokines produced by μMT cells than that by WT cells (Figure 4), suggesting that cytokine induction by myco+ exosomes is largely B cell-dependent. Compared with untreated control, small amounts of cytokines were still induced in μMT splenocytes culture, indicating that in the absence of B cells, other cell type(s) also respond to myco+ exosomes, but to a much lower level.

### B cells are the major IL-10 producers following myco+ exosome treatment

**Figure 5:**
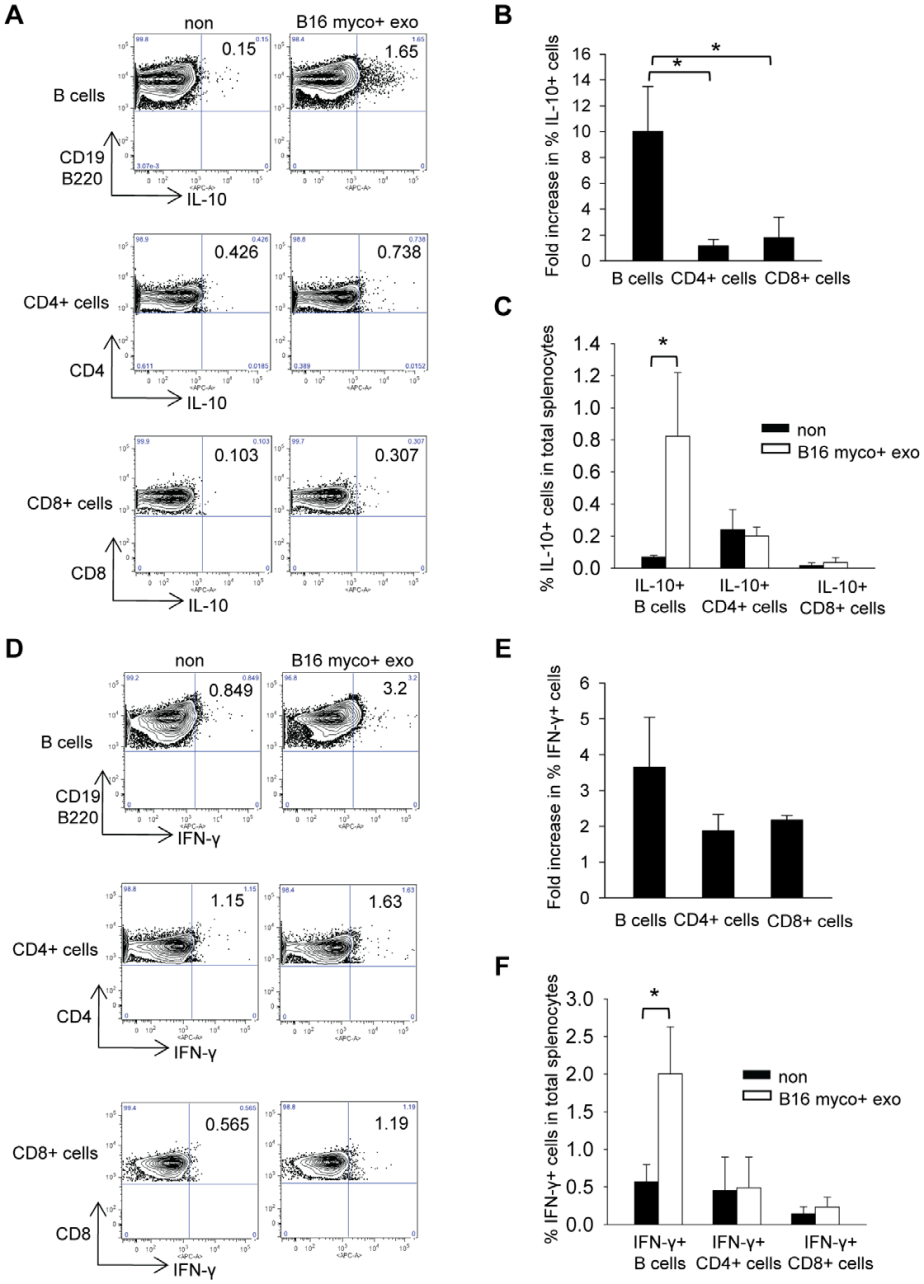
Intracellular cytokine staining of myco+ exosome-treated splenocytes. WT splenocytes were cultured with or without 1 µg/ml of B16 myco+ exosome for 48 hr in 24-well-plate at 5×10^6^ cells/1.5 ml media/well with 30 U/ml of rmIL-2. Brefeldin A was added to the culture for the last 6 hr before cells were harvested. Cells were first surface stained for CD19, B220, CD4 and CD8, and then stained for intracellular IL-10 and IFN-γ. (A) Percentage of IL-10+ cells in the B cell, CD4+ T cell and CD8+ T cell gates. Numbers in each plot represent % cells in each cell gate. Figures show the data of one representative experiment of three with similar results. (B) Fold increase of % IL-10+ cell in the B cell, CD4+ cell and CD8+ cell gate. Data represents the mean ± SD of three independent experiments. Significance at: *, P<0.05. (C) Percentage of IL-10+ B cells, IL-10+ CD4+ cells and IL-10+ CD8+ cells in total splenocytes in untreated or B16 myco+ exosome-treated splenocytes. Data represents the mean ± SD of three independent experiments. Significance at: *, P<0.05. (D) Percentage of IFN-γ+ cells in the B cell, CD4+ T cell and CD8+ T cell gates. Numbers in each plot represent % cells in each cell gate. Figures show the data of one representative experiment of three with similar results. (E) Fold increase of % IFN-γ+ cell in the B cell, CD4+ cell and CD8+ cell gate. Data represents the mean ± SD of three independent experiments. (F) Percentage of IFN-γ+ B cells, IFN-γ+ CD4+ cells and IFN-γ+ CD8+ cells in total splenocytes in untreated or B16 myco+ exosome-treated splenocytes. Data represents the mean ± SD of three independent experiments. Significance at: *, P<0.05.

To identify the major cytokine-producing cells induced by myco+ exosomes, the percentages of IL-10+ cells and IFN-γ+ cells in both B and T cell gates were analyzed 48 hr after exosome treatment by intracellular cytokine staining. The increase of % IL-10+ cells in the B cell gate upon myco+ exosome treatment was significantly higher than that in the CD4+ T cell gate or the CD8+ T cell gate (Figure 5A–B). In addition, compared with untreated control, the percentage of IL-10+ B cells in total splenocytes, but not IL-10+CD4+ or IL-10+CD8+ T cells, was significantly increased after myco+ exosome treatment (Figure 5C). There was also a greater induction of IFN-γ+ cells in the B cell gate than in the CD4+ or CD8+ T cell gate (Figure 5D–E) and the percentage of IFN-γ+ B cells in total splenocytes was significantly elevated after myco+ exosome treatment (Figure 5F). These results demonstrate that IL-10-producing B cells were preferentially induced by myco+ exosome and there was also a greater induction of IFN-γ-producing B cells than IFN-γ-producing CD4+ or CD8+ T cells.

### Induction of IFN-γ-producing T cells by myco+ exosomes is increased in the absence of B cells

**Figure 6:**
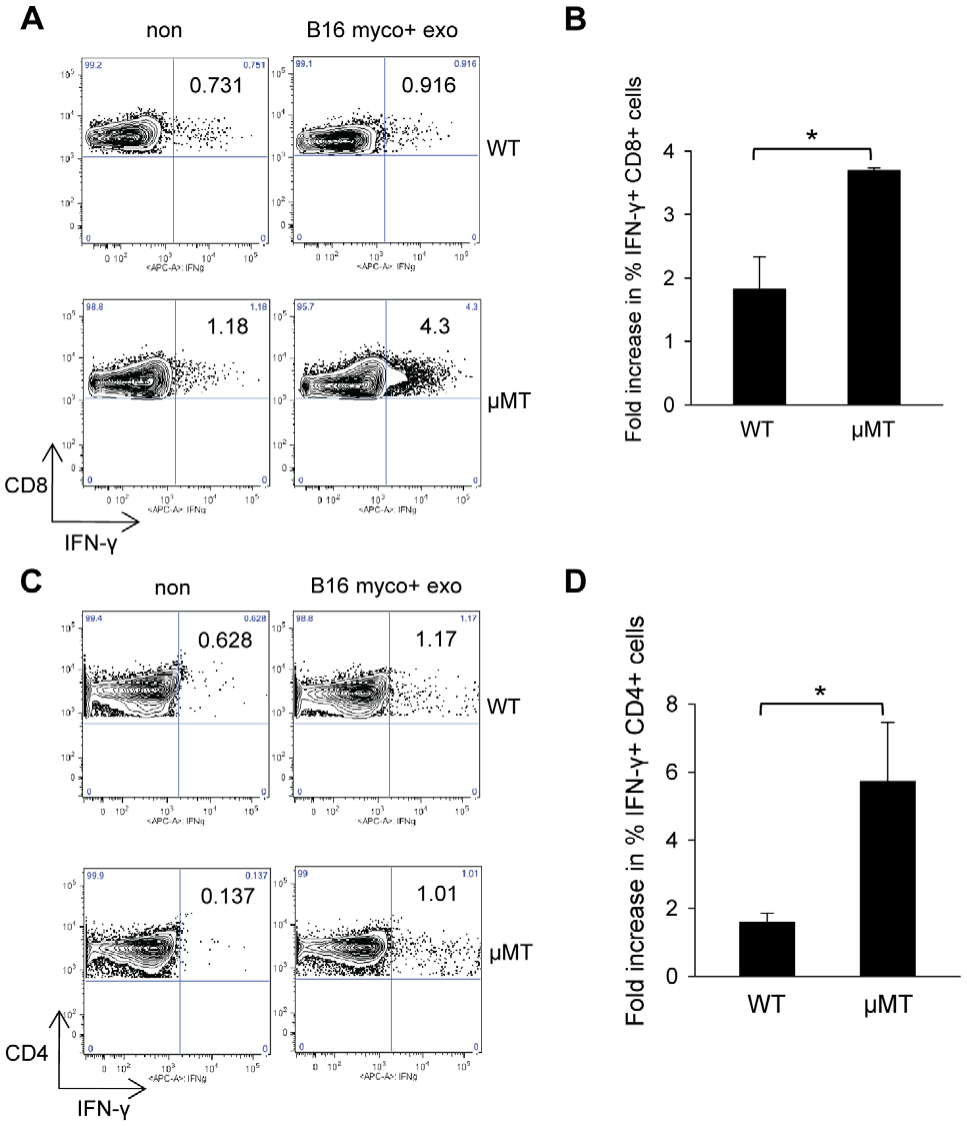
The induction of IFN-γ-producing T cells by myco+ exosomes increases in the absence of B cells. WT or μMT spleen cells were cultured with or without 1 µg/ml of B16 myco+ exosome for 48 hr and stained for intracellular IFN-γ. (A) Induction of IFN-γ+CD8+ T cells in WT and μMT splenocyte cultures. Data shows one representative experiment of three with similar results. Numbers in each plot represent % cells in CD8+ cell gate. (B) Fold increase of % IFN-γ+ cells in the CD8+ cell gate in WT and μMT splenocytes upon B16 myco+ exosome treatment. Data shows the mean ± SD of three independent experiments. Significance at: *, P<0.05. (C) Induction of IFN-γ+CD4+ T cells in WT and μMT splenocyte cultures. Data shows one representative experiment of three with similar results. Numbers in each plot represent % cells in the CD4+ cell gate. (D) Fold increase of % IFN-γ+ cells in the CD4+ cell gate in WT and μMT splenocytes upon B16 myco+ exosome treatment. Data shows the mean ± SD of three independent experiments. Significance at: *, P<0.05.

Given that myco+ exosomes induce B cell-dependent anti-inflammatory cytokine IL-10, we compared the induction of IFN-γ-producing T cells by myco+ exosomes in μMT spleen cells with that in WT spleen cells. Interestingly, in the CD8+ T cell gate the increase in the percentage of IFN-γ+ cells was significantly higher in μMT splenocytes (>3-fold) than that in WT splenocytes (<2-fold) (Figure 6A–B). Similarly in the CD4+ T cell gate, there was a greater increase in the percentage of IFN-γ+ cells in μMT splenocytes (>5-fold) compared with WT splenocytes (<2-fold) (Figure 6C–D). This suggests that the presence of myco+ exosome-activated B cells inhibits the induction of IFN-γ-producing T cells by myco+ exosome treatment.

### T cell proliferation is inhibited in the presence of myco+ exosome-treated B cells

**Figure 7:**
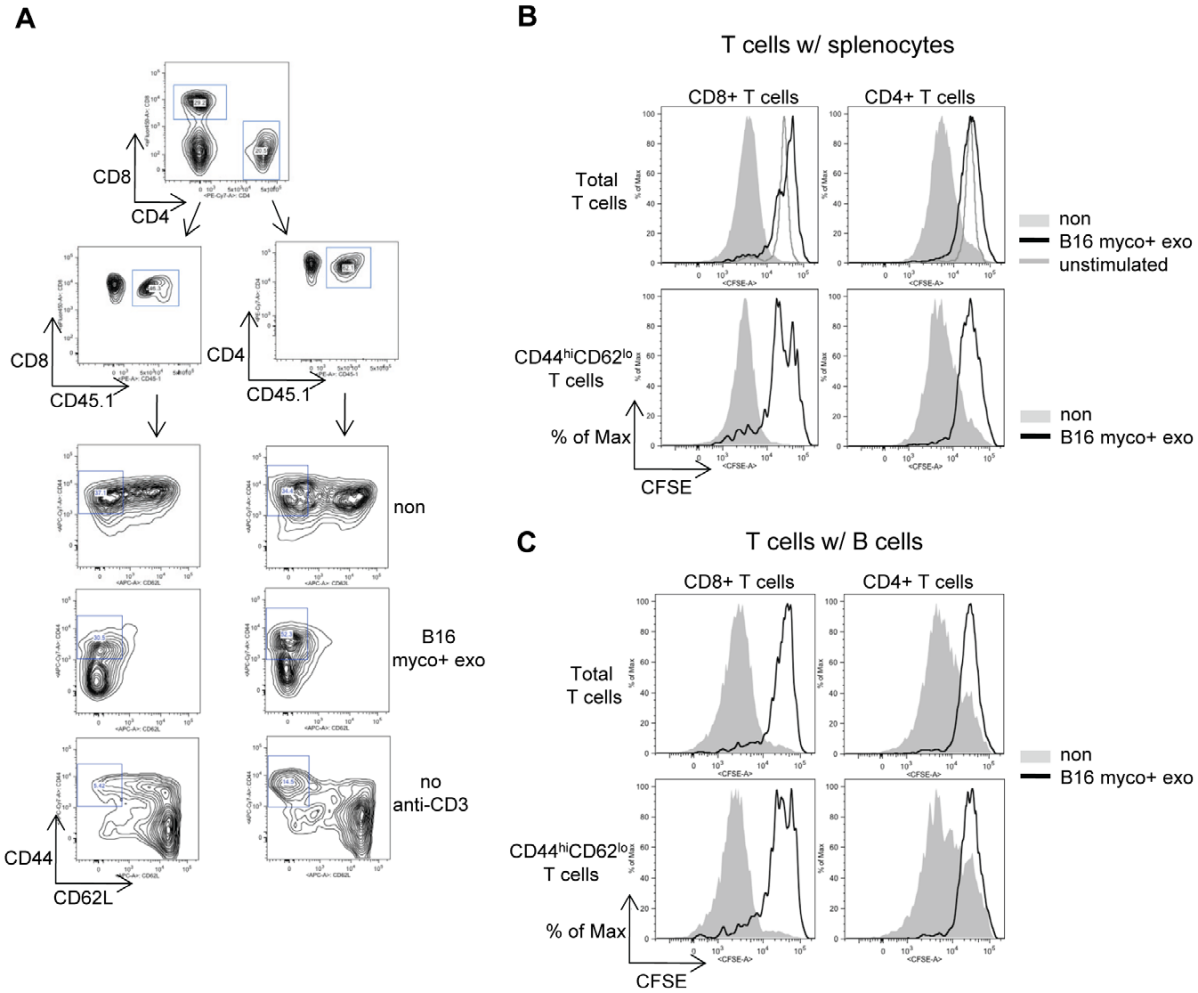
T cell proliferation is inhibited when co-cultured with myco+ exosome-treated splenocytes or purified B cells. Splenocytes (T cell-depleted) or purified splenic B cells were cultured in 24-well-plate at 2.5×10^6^ cells/well with or without 1 µg/ml of B16 myco+ exosomes for 24 hr, then 0.5×10^6^ of CFSE-labeled T cells (CD45.1+) were added to the culture and stimulated with 10 µg/ml of anti-CD3e. Cells were co-cultured for another 3 days and T cell proliferation was analyzed by CFSE dilution. (A) Gating of CD45.1+CD8+ T cells and CD45.1+CD4+ T cells. Expression of CD44 and CD62L were shown within each T cell gate in non-treated and B16 myco+ exosome treated co-cultures. Non-treated cells without anti-CD3e were included as an unstimulated control. T cells that are CD44^high^CD62L^low^ represent the activated T cell subset. (B) Proliferation of CD8+ T cells and CD4+ T cells in myco+ exosome-treated splenocytes shown by CFSE dilution. Total T cells: total CD8+ or CD4+ T cells. CD44^hi^CD62L^lo^ T cells: T cell subsets that are CD44^high^CD62L^low^. Unstimulated: Non-treated T cells without anti-CD3 stimulation. (C) Proliferation of CD8+ T cells and CD4+ T cells when co-cultured with myco+ exosome-treated B cells, shown by CFSE dilution.

Since IL-10 production by B cells was dramatically induced upon myco+ exosome treatment and IL-10 is known to negatively regulate T cell activity, we examined if T cell proliferation was affected by myco+ exosome-activated B cells. Anti-CD3 stimulated T cell proliferation was first examined in splencoyte culture. Purified T cells expressing the congenic marker CD45.1 were labeled with CFSE and co-cultured with T cell-depleted, myco+ exosome pre-treated splenocytes. T cells were stimulated with 10 µg/ml of anti-mouse CD3e and were allowed to proliferate for 3 days. T cell proliferation was demonstrated by CFSE dilution. Notably, the proliferation of CD8+ T cells and CD4+ T cells were both inhibited when the T cells were co-cultured with myco+ exosome-treated splenocytes, compared with untreated splenocytes. Proliferation of T cells with the CD44^hi^CD62L^lo^ phenotype, representing the activated T cell subset, also was found inhibited (Figure 7A–B).

Next, we tested if B cells alone, upon myco+ exosomes treatment, can inhibit T cell proliferation. B cells were purified from total splenocytes by MACS depletion of T cells and Non-B-APCs. T cells were co-cultured with myco+ exosome pre-treated B cells and stimulated with anti-CD3. CFSE dilution demonstrated that myco+ exosome-treated B cells were equally capable of inhibiting the proliferation of total CD8+ T cells and CD4+ T cells, as well as T cells with the CD44^hi^CD62L^lo^ phenotype (Figure 7C).

### TCR signaling is impaired in myco+ exosome-treated splenocytes

**Figure 8:**
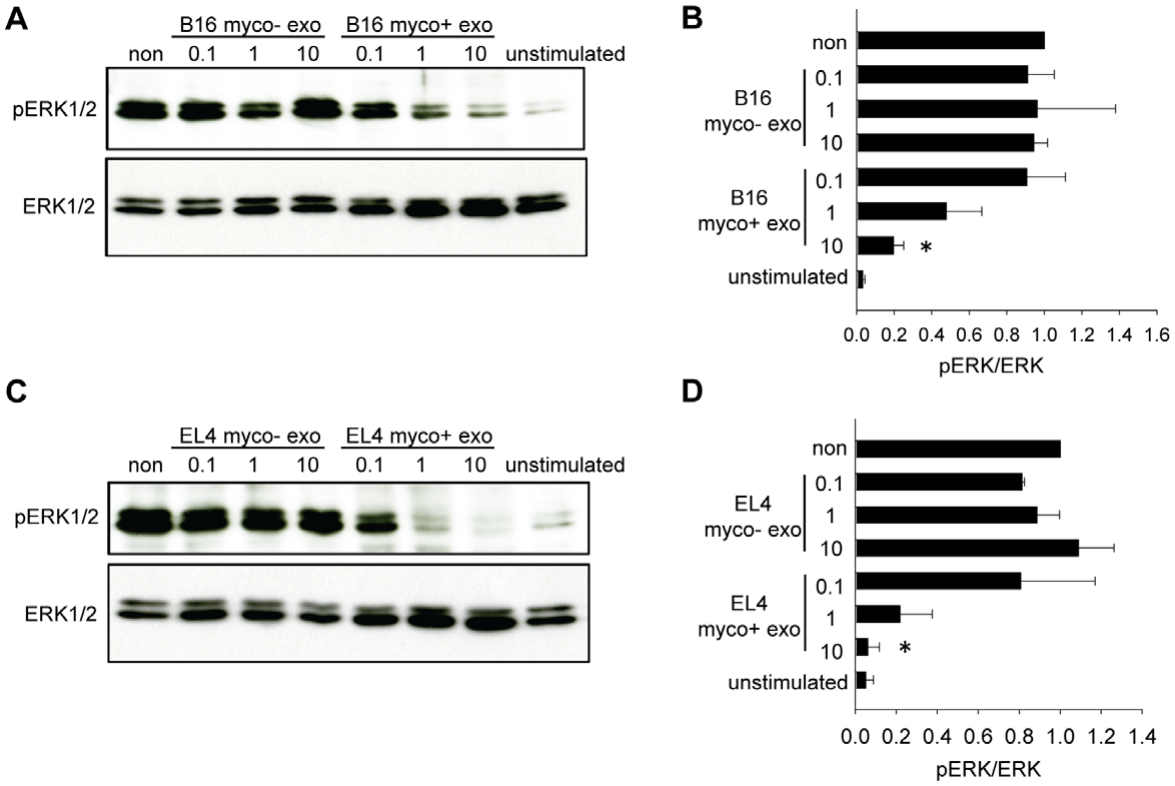
Myco+ exosome treatment inhibits anti-CD3-stimulated ERK phosphorylation. Splenocytes were treated with 0.1, 1 or 10 µg/ml of myco+ exosomes or myco− exosomes for 48 hr, then 1 µg/ml of anti-CD3e were added for 30 min. Cells were prepared for western blot analysis using antibodies against phosphorylated ERK protein (pERK1/2) and total ERK protein (ERK1/2). (A) Representative western blot figures of splenocytes upon B16 myco− exosome or B16 myco+ exosome treatment. Non: non-treated splenocytes with anti-CD3 stimulation. Unstimulated: non-treated splenocytes cells without anti-CD3 stimulation. (B) Relative expression of pERK normalized to total ERK. Data represents the mean ± SD of three independent experiments. 10 µg/ml of B16 myco+ exosome treatment significantly decreased pERK/ERK compared with non treatment. Significance at: *, P<0.05. (C) Representative western blot figures of splenocytes upon EL4 myco− exosome or EL4 myco+ exosome treatment. (D) Relative expression of pERK normalized to total ERK. Data represents the mean ± SD of three independent experiments. 10 µg/ml of EL4 myco+ exosome treatment significantly decreased pERK/ERK compared with non treatment. Significance at: *, P<0.05.

We next examined whether anti-CD3-stimulated T cell receptor (TCR) signaling was impaired in myco+ exosomes-treated splenocytes. CD3 cross-linking triggers several downstream signal transduction pathways that lead to T cell activation and proliferation, including the MAP kinase pathway. Thus ERK phosphorylation, the last step in the MAP kinase cascade, was examined upon anti-CD3 stimulation. Splenocytes were treated with increasing doses of exosomes (0.1, 1 and 10 µg/ml) or cultured untreated for 48 hrs, and then stimulated with 1 µg/ml of anti-CD3e for 30 min before being harvested. Phosphorylation of ERK proteins (pERK1/2) was examined by Western blotting. Robust ERK phosphorylation was detected in untreated splenocytes and splenocytes treated with myco− exosome, whereas in splenocytes treated with myco+ exosomes, ERK phosphorylation was significantly reduced in a dose-dependent manner (Figure 8).

### Cytokine induction by myco+ exosomes does not require exosome membrane integrity

**Figure 9:**
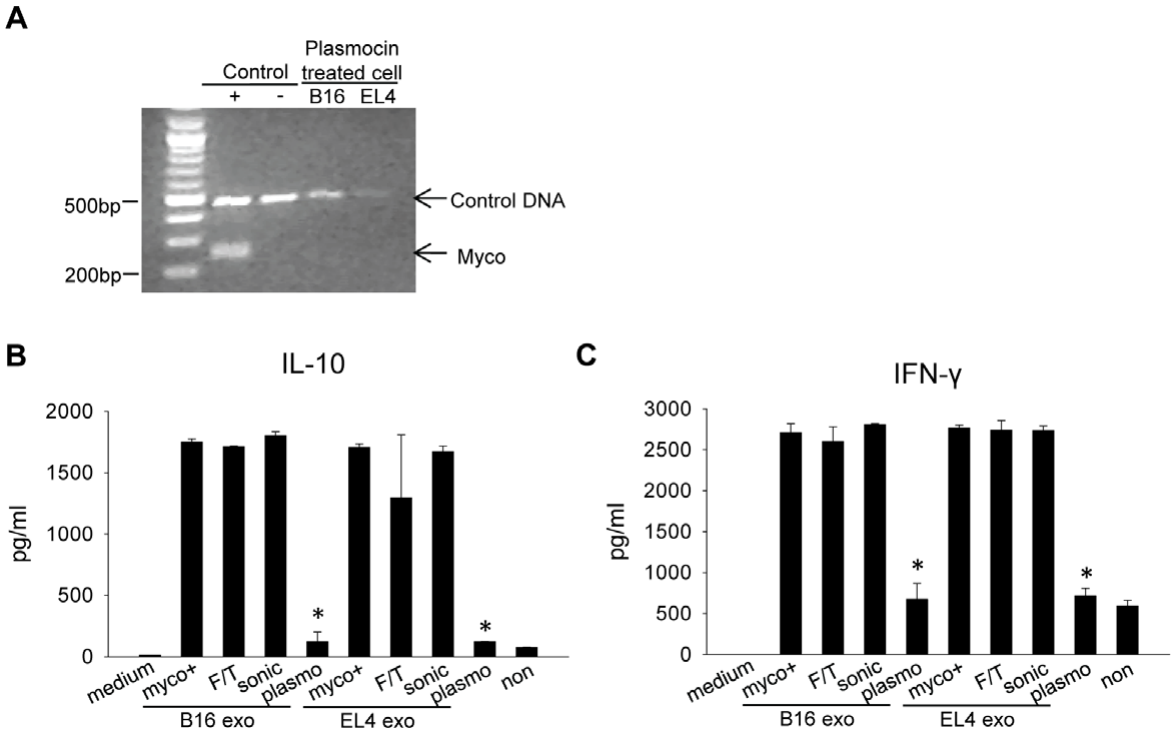
Cytokine induction by myco+ exosomes after exosome membrane disruption or mycoplasma removal reagent treatment of parental cells. (A) Mycoplasma-infected cells were treated with Plasmocin for 2 wk and tested to be mycoplasma-free. (B) B16 and EL4 myco+ exosomes were subjected to 5 cycles of freeze/thaw (F/T) or sonication (sonic). Splenocytes were treated with 1 µg/ml of myco+ exosomes, F/T exosomes, sonic exosomes or exosomes derived from Plasmocin-treated cells (plasmo) for 72 hr. IL-10 production was measured by ELISA. (C) IFN-γ production measured by ELISA. Induction of IL-10 and IFN-γ by plasmo exosomes was significantly reduced compared with intact, F/T and sonic exosomes. Significance at: *, P<0.05.

To determine if intact exosome structure is required for the stimulatory effect, myco+ exosome were subjected to 5 cycles of freeze/thaw or repeated sonication, which has been shown to disrupt exosome membrane [5]. Interestingly, membrane disruption had little impact on the cytokine induction (Figure 9B–C) and B cell activation (data not shown) effects of myco+ exosomes, whereas exosomes derived from cells whose mycoplasma infection had been removed by Plasmocin completely lost their stimulatory ability. Since the ultra-filtration process during exosome preparation excluded the whole mycoplasma organisms, these results suggest that the stimulatory effect of myo+ exosomes was due to mycoplasma-derived components incorporated into exosomes but does not require exosome membrane integrity.

## Discussion

Mycoplasmas interact with host cells by different ways such as adherence, invasion or fusion. Following infection, they are potent modulators of the host immune systems. We have observed that mycoplasmas can also indirectly affect immune cells by modifying exosomes released by host tumor cells with specific immunoregulatory properties. In this study, we demonstrate that tumor cells with mycoplasma infection release exosomes with B cell stimulatory and cytokine induction ability, which were not observed in exosomes released from uninfected tumor cells. Moreover, B cells activated by these exosomes were capable of inhibiting T cell responses. These effects of exosomes exclusively correlated with the infection status of their parental cells, as the effects were completely abolished after treating the infected cells with mycoplasma removal reagent.

It has been reported that macrophages infected with intracellular pathogens such as *Mycobacterium tuberculosis* and *Mycobacterium bovis* BCG release exosomes that contain pathogen-associated molecular patterns, and these exosomes are able to stimulate a proinflammatory response both *in vitro* and *in vivo* [15]. It was also reported that mycobaterial components actively traffic within infected macrophages with access to the MVB pathway and are released in exosome-like extracellular vesicles [16], [42]. In addition, DCs infected with mycoplasmas give rise to exosome preparations which are able to induce polyclonal B cell mitogenisis [17]. In fact, the ability of mycoplasmas to invade non-phagocytic host cells and enter the endosome pathway [43] make exosomes produced by non-phagocytic cells including tumor cells equally susceptible targets for the incorporation of pathogen components. Nevertheless, there remains a possibility that mycoplasmas could be co-isolated with exosomes, and whether whole, viable mycoplasmas are co-isolated with exosomes largely depends on the stringency of excluding mycoplasma-sized particles during exosome purification. In our study, electron microscopy showed that whole mycoplasma organisms were absent in the exosomes prepared from infected cell lines. Those exosome preparations induced splenic B cell activation and cytokine production, typically seen within 48–72 h after treatment. Such effect was not affected by repeated freeze-thaw cycles or sonication of the exosome preparation, suggesting that intact exosome membrane structures are not necessary to initiate the immune response.

Mycoplasmas contain abundant lipoproteins, many of which are immunogenic and/or mitogenic. Certain lipoproteins were found to induce T cell-independent B cell blastogenesis and secretion of proinflammatory cytokines [44], [45], [46], resembling the effect of myco+ exosomes we observed. Many mycoplasmal B cell mitogens function through a pathway distinct from that of lipopolysaccharide (LPS) [20], [44]. In an effort to identify the potential mycoplasma ligand(s) in exosomes responsible for the responses, proteomic analysis was performed on both myco+ and myco− exosomes. Mass spectrometry analysis on both myco+ B16 exosomes and myco− B16 exosomes identified a group of membrane associated proteins and lipoproteins with potential pro-inflammatory properties that are specifically present on myco+ exosomes (Table S1). However, other mycoplasma components, such as glycan moieties or lipids, may also contribute to the B cell stimulatory activity [32]. Additionally, mycoplasma infection also seems to alter the endogenous protein composition of tumor-derived exosomes, as a wide variety of proteins including membrane proteins, enzymes, chaperons, nuclear proteins and structural proteins were found at a higher level in myco+ B16 exosome (Table S2), while another large repertoire of proteins were found down-regulated in myco+ B16 exosomes (Table S3). The immune responses stimulated by exosome-incorporated mycoplasma components could potentially interfere with the intrinsic immunomodulatory properties of exosomes, and it is possible that exosomes released from mycoplasma-infected cells could stimulate similar immune responses regardless of the type of host cells.

The anti-inflammatory cytokine IL-10 was found expressed at a higher level in certain mycoplasma-associated human diseases [34]. Here in murine splenocytes culture, we found that myco+ exosomes predominantly induce IL-10 in addition to IFN-γ and the production of these cytokines was largely B cell-dependent. Moreover, the IL-10-producing cells were mainly induced in the B cell population, not in the T cell population (Figure 5). These results emphasize a role of B cells in producing anti-inflammatory cytokines, especially IL-10, in response to exosomes derived from mycoplasma-infected cells.

It has been reported that B cell-derived IL-10 can be produced by both naïve and memory B cells, as well as the regulatory B cell subset with a CD1d+CD5+ phenotype [47]. Although the exact B cell subset(s) producing IL-10 in response to myco+ exosomes is not clear, it is likely that more than one subset contributed to the production of IL-10. IL-10 sustains the growth of activated B cells and acts as a hinge cytokine by suppressing cell mediated immunity while promoting humoral immunity [48], [49]. B cell-derived IL-10 can function in the prevention of inflammatory responses in autoimmune diseases as well as in the down-regulation of active disease exacerbation [47]. Our observation that myco+ exosome-treated μMT spleen cells produce dramatically decreased IL-10 while having significantly increased percentage of IFN-γ-producing T cells, suggests that T cells are better activated in the absence of B cells and that myco+ exosome-activated B cells can potently suppress T cell activity.

The inhibitory effect of myco+ exosome-activated B cells on T cells was further demonstrated by T cell proliferation assay. Anti-CD3-stimulated proliferation of both CD4+ T cells and CD8+ T cells was strongly inhibited by myco+ exosome-treated B cells (Figure 7). Such inhibition correlated with impaired TCR signaling in response to anti-CD3 stimulation (Figure 8). Presumably B cell-derived IL-10 and/or the IL-2 deprivation by B cells with up-regulated CD25 could be responsible for the inhibitory effect on T cells.

The impact of mycoplasma infection of tumor cells on tumor-associated immune responses remains unclear. Certain mycoplasma proteins have been shown to promote cancer cell invasiveness and metastasis both *in vitro* and *in vivo* [50]. Our observation provides implications of immune modulation by co-existing opportunistic pathogens in tumor-bearing hosts. Our studies identify exosomes as effective vehicles for intracellular pathogens to communicate indirectly with immune cells to confer their immunomodulatory effects. Our results also suggest that mycoplasmas infecting tumor cells could utilize tumor-derived exosomes to induce a B cell response and the production of B cell-derived regulatory cytokines IL-10, which could further lead to the inhibition of T cell activity. Such effect may not only diminish the inflammatory response directed against these pathogens, but also jeopardize effective T cell responses in anti-tumor immunity.

In conclusion, our study characterizes the splenic B cell and T cell responses to exosomes derived from tumor cells with mycoplasma infection. We demonstrate the preferential activation of B cells and B cell-dependent cytokine induction by these exosomes and the subsequent inhibition of T cell proliferation and TCR signaling. Our results dissect the reactions of B and T lymphocytes in response to tumor-derived exosomes carrying mycoplasma components and reveal the potential antagonizing effect of B cell activation to T cell activity. These observations will help us better understand the impact of pathogenic components released in the form of exosomes on host immune modulation.

## Materials and Methods

### Cell lines and mice

Murine B16 and EL4 cell lines were originally purchased from American Type Culture Collection. Cells were cultured in RPMI 1640 supplemented with 10% FBS, 2 mM L-glutamine, 0.1 mM non-essential amino acids, 1 mM sodium pyruvate, 10 mM HEPES, Antibiotic-Antimicotic (GIBCO), and 50 µM β-mercaptoethanol. Female C57BL/6J (CD45.2+) mice, μMT (Ighm*^tm1Cgn^*) mice and the congenic CD45.1+ B6 (B6.SJL-*Ptprc^a^Pep3^b^*/BoyJ) mice were purchased from the Jackson Laboratory. Animals were maintained in a pathogen-free animal facility at University of Pittsburgh Biotechnology Center. All animal-related experiments were conducted in strict accordance with the guidelines for the care and use of Laboratory Animals of the National Institutes of Health and animal protocol 0804421B-1 was approved by the University of Pittsburgh Institutional Animal Care and Use Committee, assurance number A3187-01. Mice were euthanized in CO_2_ tank for organ harvesting.

### Mycoplasma detection and elimination

Cell lines were screened for mycoplasma using MycoAlert™ mycoplasma detection kit (Lonza) and infections were confirmed using LookOut® Mycoplasma PCR detection kit (Sigma). DNA was separated in a 1.2% agarose gel and stained with ethidium bromide. For mycoplasma removal, infected cell lines were treated with Plasmocin™ (Invivogen) for 2 weeks and then cultured for another week before PCR test to ensure complete elimination.

### Exosome purification

Exosomes were isolated from cell culture supernatant by differential centrifugation and filtration. FBS used for culture media was pre-cleared by ultracentrifugation at 100,000× g for 3 hr at 4°C. 48 hr culture supernatants were centrifuged at 1000× g for 10 min and 10,000× g for 30 min to remove cell and membrane debris, then filtered through 0.22 µm sterilizing filter (Corning), and further concentrated using Centricon Plus-70 100 kD cutoff filter units (Millipore). The concentrated supernatants were subjected to ultracentrifugation at 100,000× g for 1 hr. Exosomes pellets were washed with sterile PBS, centrifuged at 100,000× g for 1 hr, and resuspended in sterile PBS. Exosome quantification was done by Bradford protein assay (Bio-Rad).

### Transmission electron microscopy

Exosome preparations were loaded on Formvar/carbon-coated grids and negatively stained with 1% uranyl acetate. Pictures were taken on a JEM-1011 transmission electron microscope with the Advanced Microscopy Techniques (AMT) software.

### Splenocytes culture

Spleens were isolated from mice euthanized in CO_2_ tank. Single cell suspensions were prepared by mincing the tissues through a 70 µm cell strainer. Erythrocytes were depleted using ACK cell lysing buffer (Biowhittaker). Splenocytes were cultured in complete RPMI 1640 media, in the presence of 30 U/ml recombinant murine IL-2 (Biolegend).

### ELISA

IL-10 level in culture supernatants was detected using mouse IL-10 ELISA kit (eBioscience). IFN-γ ELISA was performed using purified anti-mouse IFN-γ as capture antibody and biotinylated anti-mouse IFN-γ as detection antibody (BD Pharmingen).

### Flow cytometry

For surface staining, cells were washed in staining buffer (2% FBS, 0.4% NaN3 and 1 mM EDTA in PBS) and stained with Ethidium monoazide (EMA) for dead cell exclusion. Cells were then washed and incubated with purified anti-mouse CD16/32 (Fc-block, eBiosciences) for 10 min on ice, followed by incubation with fluorochrome-conjugated antibodies for 30 min on ice. When biotinylated antibodies were used, cells were further incubated with secondary reagent (streptavidin-fluorochrome). For intracellular cytokine staining, cells were treated with Brefeldin A for the last 6 hrs in culture before being harvested. After surface staining, cells were fixed and permeablized with Fix/Perm solution (BD Biosciences) and then stained with cytokine antibodies in Perm/Wash buffer (BD Biosciences) for 1 hr at RT or overnight at 4°C in dark. Antibodies used for surface marker characterization include: APC-eFluor780-B220, PacificBlue-CD19, PE-Cy7-CD25, PE-CD40, FITC-CD86, PE-CD80, PE-Cy7-CD23, FITC-CD19, eFluor450-IgD, PE-CD1d, PE-CD43, PE-CD8, APC-eFluor780-CD4, FITC-CD69, APC-CD62L, eFluor450-CD44, PE-7-CD4, APC-CD8 and Biotin-CD5 from eBiosciences, and FITC-IgM from BD Bioscience. Antibodies used for intracellular cytokine characterization include: FITC-CD19, FITC-B220, APC-eFluor780-CD4, PacificBlue/eFluor450-CD8, APC-IL-10 and APC-IFN-γ from eBiosciences. Secondary reagents used include streptavidin-APC-eFluor780 and streptavidin-APC-Cy7 from eBiosciences. Antibodies and secondary reagent used for T cell proliferation assays include: PE-Cy7-CD4, eFluor450-CD8, PE-CD45.1, APC-CD62L, Biotin-CD44 and streptavidin-APC-Cy7 from eBiosciences. Flow acquisition was performed on LSRII analyzers (BD Biosciences) and data were analyzed using the Flowjo software (Tree star Corp.).

### T cell proliferation assay

Splenic single cell suspension was prepared from C57BL/6 (CD45.2+) mice as mentioned above. For T cell depletion, splenocytes were first incubated with biotin-anti-mouse CD3 (10 µl Ab/100×10^6^ cells/1 ml, eBioscience) at 4°C for 15 min, then with streptavidin MACS beads (100 µl/100×10^6^ cells/1 ml, Miltenyi) at 4°C for 15 min, followed by negative selection using autoMACS™ Pro Separator (Miltenyi). To purify B cells from total splenocytes, cells were first incubated with biotin anti-mouse CD3, CD11c, F4/80 and PDCA-1(each at 10 µl Ab/100×10^6^ cells/1 ml, ebioscience), then with streptavidin MACS beads, followed by autoMACS negative selection. B cell purity was checked by FACS and the percentages of remaining Non-B-APCs are: CD11c+ cells <1.3%; F4/80+ cells <0.1%; and PDCA-1+ cells <1%. To purify T cells from CD45.1+ B6 splenocytes, cells were first incubated with biotin-anti-mouse CD19, B220, IgM, CD11c, F4/80, PDCA-1, IA/IE, and CD25 (eBioscience), then with streptavidin MACS beads, followed by autoMACS negative selection. T cell purity was checked by FACS and the percentage of CD4+ plus CD8+ T cells reached 90%. Purified T cells were labeled with 2 µM of CFSE. To assess anti-CD3-stimulated T cell proliferation, T cell-depleted splenocytes or purified B cells were cultured in 24-well-plate at a cell density of 2.5×10^6^/1 ml media/well, with or without treatment of 1 µg/ml B16 myco+ exosomes. On the following day, 0.5×10^6^ of purified CD45.1+ T cells were added to each well and the media volume was brought up to 3 ml. 10 µg/ml of purified anti-mouse CD3e (BD Pharmingen) was added for stimulation. Cells were harvested after 3 days and the CFSE dilution of CD45.1+ T cells were analyzed by FACS.

### CFSE labeling

T cells purified from CD45.1+ splenocytes were labeled with CFSE using CellTrace™ CFSE cell proliferation kit (Molecular Probes, Invitrogen). Briefly, 2 µM CFSE working solution were prepared in PBS containing DMSO (10%), and mixed well with cell pellet at the ratio of 5×10^6^ cells/1 ml of CFSE. Cells were incubated at 37°C for 10 min and the reaction was quenched with complete media. Cells were then washed in warm PBS.

### Western blot

Splenocytes were collected after treatment and lysed in NP-40 lysis buffer in the presence of protease inhibitor (Sigma-Aldrich) and phosphotase inhibitor (1 mM Na_2_VO_4_). 10 µg of cell lysate was separated on 12% SDS-PAGE and transferred onto polyvinylidene difluoride membranes (Millipore). The membrane was blocked and incubated with phosphor-p44/42 MAP kinase antibody (Cell Signaling), followed by horseradish peroxidase-conjugated anti-rabbit secondary antibody (Santa-Cruz). Protein bands were visualized using an enhanced chemiluminescence detection kit (PerkinElmer Life Science). To blot for total ERK protein, the same membrane was stripped in stripping buffer (Pierce), blocked, incubated with p44/42 MAP antibody (Cell Signaling), and followed by anti-rabbit secondary antibody. Densitometric quantitations were done using the ImageJ software.

### Statistics

Statistical analysis was performed by Student's *t*-test. A value of P<0.05 was considered as statistically significant.

### Mass spectrometry

The LC-MS/MS and database searching were performed by the Mass Spectrometry Platform of Cancer Biomarkers Facility in University of Pittsburgh Cancer Institute. Briefly, 10 µg of total cell lysate was resolved by 1D-PAGE and subjected to in-gel digestion. Tryptic peptides were extracted, lyophilized and resuspended in 0.1% trifluoroacetic acid. Nanoflow reversed-phase liquid chromatography (RPLC) was performed using a Dionex Ultimate 3000 LC system (Dionex Corporation, Sunnyvale, CA) coupled online to a linear ion trap (LIT) mass spectrometer (LTQ, ThermoFisher Scientific, San Jose, CA). The LIT-MS was operated in a data dependent MS/MS mode in which each full MS scan was followed by seven MS/MS scans where the seven most abundant peptide molecular ions are selected for collision-induced dissociation (CID). Tandem mass spectra were searched against a combined UniProt mouse protein database (03/2010) from the European Bioinformatics Institute (http://www.ebi.ac.uk/integr8) using SEQUEST (ThermoFisher Scientific). In addition, data were searched against two combined UniProt mycoplasma database (*M. hominis*/*A. laidlawii*; and *M. agalactiae/arthriditis/pneumoniae/pulmonis*). Results from both searches were further filtered using software developed in-house to determine unique peptides and proteins.

## Supporting Information

Table S1 **Data show the selective search results against two combined UniProt mycoplasma database: M. hominis/A. laidlawii, and M. agalactiae/arthriditis/pneumoniae/pulmonis.**
(DOCX)

Table S2 **Data show the selective search results against a combined UniProt mouse protein database (03/2010) from the European Bioinformatics institute (** http://www.ebi.ac.uk/integr8 **).**
(DOCX)

Table S3 **Data show the selective search results against a combined UniProt mouse protein database (03/2010) from the European Bioinformatics institute (** http://www.ebi.ac.uk/integr8 **).**
(DOCX)
