# Supplementary material for: Exosomes Released from Mycoplasma Infected Tumor Cells Activate Inhibitory B Cells
Source: PLoS One. 2012 Apr 27;7(4):e36138. doi: 10.1371/journal.pone.0036138 (PMC3338602; doi:10.1371/journal.pone.0036138)
Supplement: Table S1 — Data show the selective search results against two combined UniProt mycoplasma database: M. hominis/A. laidlawii, and M. agalactiae/arthriditis/pneumoniae/pulmonis. (DOCX) [file pone.0036138.s001.docx]

Table S1

Mycoplasma-associated proteins specifically identified in myco+ B16 exosomes

| **Proteins** | **Accession number** | | **Peptide number** | **% in total peptides** |
| --- | --- | --- | --- | --- |
| **Lipoproteins and membrane proteins** | |  |  |  |
| Lmp3 protein | | D1J7V1 | 3 | 0.67 |
| Integral membrane protein | | A9NE35 | 2 | 0.45 |
| P37-like (*M.hyorhinis*) ABC transporter substrate-binding lipoprotein | | D1J8E9 | 1 | 0.22 |
| P75 protein | | D1J8G0 | 1 | 0.22 |
| Lipoprotein (*M.pulmonis*) | | Q98PI7 | 5 | 1.3 |
| Lipoprotein (*M.pulmonis*) | | Q98R04 | 2 | 0.52 |
| Lipoprotein (*M.pulmonis*) | | Q98RF3 | 2 | 0.52 |
| Massive surface protein MspC (*M.arthritidis*) | | B3PMQ9 | 2 | 0.52 |
| Massive surface protein MspH (*M.arthritidis*) | | B3PN22 | 2 | 0.52 |
| **Enzymes** | |  |  |  |
| DNA methylase | | A9NH63 | 6 | 1.34 |
| Glucose-6-phosphate 1-dehydrogenase | | A9NGU2 | 5 | 1.12 |
| Peptidase M3B, oligoendopeptidase F | | A9NH01 | 3 | 0.67 |
| DNA polymerase III, gamma and tau subunit | | A9NE33 | 2 | 0.45 |
| Excinuclease ABC, subunit C | | A9NEZ8 | 2 | 0.45 |
| V-type H+-transporting ATPase, subunit D | | A9NHD6 | 2 | 0.45 |
| Acetyl-CoA carboxylase, biotin carboxylase | | A9NFE7 | 2 | 0.45 |
| DNA polymerase III beta chain | | D1J7E2 | 2 | 0.45 |
| NAD(+)-dependent DNA ligase | | D1J7Y7 | 2 | 0.45 |
| DNA-directed RNA polymerase subunit alpha | | Q98Q08 | 5 | 1.13 |
| ATP synthase epsilon chain | | Q98QU6 | 4 | 1.04 |
| Glucose-6-phosphate isomerase | | P78033 | 4 | 1.04 |
| Oligo-1,6-Glucosidase | | Q98PT6 | 4 | 1.04 |
| Esterase/Lipase 1 | | Q98RH3 | 3 | 0.78 |
| **Others** | |  |  |  |
| 50S ribosomal protein L16 | | A9NEE0 | 2 | 0.45 |
| ABC Transporter ATP-binding protein | | D1J8A9 | 2 | 0.45 |
| Protein recA | | A9NGM3 | 2 | 0.45 |
| Transcriptional regulator, AraC family | | A9NHA0 | 2 | 0.45 |
| ABC-type transport system, permease component | | A9NH78 | 2 | 0.45 |
| Transcriptional regulator, MarR family | | A9NHK8 | 2 | 0.45 |
| Cell division protein ftsH | | D1J8L1 | 1 | 0.22 |
| Cell division protein ftsZ | | Q50318 | 3 | 0.78 |
| ABC Transporter ATP-binding protein | | B3PM05 | 3 | 0.78 |
| Chromosome replication initiation and membrane attachment protein | | B3PMZ9 | 3 | 0.78 |
| Segregation and condensation protein A | | D3VRT6 | 3 | 0.78 |
| Cytadherence high molecular weight protein 2 | | P75471 | 3 | 0.78 |
