# Supplementary material for: Exosomes Released from Mycoplasma Infected Tumor Cells Activate Inhibitory B Cells
Source: PLoS One. 2012 Apr 27;7(4):e36138. doi: 10.1371/journal.pone.0036138 (PMC3338602; doi:10.1371/journal.pone.0036138)
Supplement: Table S2 — Data show the selective search results against a combined UniProt mouse protein database (03/2010) from the European Bioinformatics institute ( http://www.ebi.ac.uk/integr8 ). (DOCX) [file pone.0036138.s002.docx]

Table S2

Endogenous murine proteins up-regulated in myco+ B16 exosomes compared with myco- B16 exosomes

| **Protein** | **Accession number** | **Peptide number** | | **% in total peptides** | |
| --- | --- | --- | --- | --- | --- |
|  |  | **Myco- Exo** | **Myco+ Exo** | **Myco- Exo** | **Myco+ Exo** |
| Basement membrane-specific heparan sulfate proteoglycan core protein | Q05793 | 2 | 10 | 0.06 | 0.40 |
| Transmembrane protein 63B | Q3TWI9 | 2 | 6 | 0.06 | 0.24 |
| Calpain-11 | Q6J756 | 0 | 5 | 0 | 0.20 |
| X-prolyl aminopeptidase (Aminopeptidase P) 2, membrane-bound | B1AVD2 | 0 | 4 | 0 | 0.16 |
| Myotubularin related protein 2 (Fragment) | B8JJF3 | 0 | 4 | 0 | 0.16 |
| Vomeronasal 1 receptor, F4 | Q05A06 | 0 | 4 | 0 | 0.16 |
| Slit homolog 3 protein | Q9WVB4 | 0 | 4 | 0 | 0.16 |
| Diamine oxidase-like protein 2 | Q6IMK7 | 0 | 3 | 0 | 0.12 |
| Keratinocyte-associated transmembrane protein 2 | Q8K201 | 0 | 3 | 0 | 0.12 |
| DNA repair protein REV1 | Q920Q2 | 0 | 3 | 0 | 0.12 |
| Aconitate hydratase, mitochondrial | Q99KI0 | 0 | 3 | 0 | 0.12 |
| Bifunctional purine biosynthesis protein PURH | Q9CWJ9 | 0 | 3 | 0 | 0.12 |
| Fascin-3 | Q9QXW4 | 0 | 3 | 0 | 0.12 |
| Collagen alpha-4(VI) chain | A2AX52 | 0 | 2 | 0 | 0.08 |
| Testis expressed gene 16 (Fragment) | B1AXV8 | 0 | 2 | 0 | 0.08 |
| Ankrd11 protein | B2RY01 | 0 | 2 | 0 | 0.08 |
| Bone morphogenetic protein receptor type-2 | O35607 | 0 | 2 | 0 | 0.08 |
| Poly [ADP-ribose] polymerase 2 | O88554 | 0 | 2 | 0 | 0.08 |
| Nuclear transition protein 2 | P11378 | 0 | 2 | 0 | 0.08 |
| Kallikrein 1-related peptidase b22 | P15948 | 0 | 2 | 0 | 0.08 |
| Isoform Mdm2-p90 of E3 ubiquitin-protein ligase Mdm2 | P23804-1 | 0 | 2 | 0 | 0.08 |
| Fibromodulin | P50608 | 0 | 2 | 0 | 0.08 |
| Neurocan core protein | P55066 | 0 | 2 | 0 | 0.08 |
| T-complex protein 1 subunit zeta | P80317 | 0 | 2 | 0 | 0.08 |
| Tubulin beta-5 chain | P99024 | 0 | 2 | 0 | 0.08 |
| Ras suppressor protein 1 | Q01730 | 0 | 2 | 0 | 0.08 |
| Cation-independent mannose-6-phosphate receptor | Q07113 | 0 | 2 | 0 | 0.08 |
| Histone H1t | Q07133 | 0 | 2 | 0 | 0.08 |
| BPAG1 isoform 3 (Fragment) | Q1KP04 | 0 | 2 | 0 | 0.08 |
| Killer cell lectin-like receptor 2 | Q60660 | 0 | 2 | 0 | 0.08 |
| AKT-interacting protein | Q64362 | 0 | 2 | 0 | 0.08 |
| All-trans-retinol 13,14-reductase | Q64FW2 | 0 | 2 | 0 | 0.08 |
| Proline-rich protein 14 | Q7TPN9 | 0 | 2 | 0 | 0.08 |
| Nardilysin | Q8BHG1 | 0 | 2 | 0 | 0.08 |
| Microtubule-associated protein 1S | Q8C052 | 0 | 2 | 0 | 0.08 |
| Condensin complex subunit 2 | Q8C156 | 0 | 2 | 0 | 0.08 |
| Transcription factor SOX-30 | Q8CGW4 | 0 | 2 | 0 | 0.08 |
| Rootletin, isoform 1 | Q8CJ40-1 | 0 | 2 | 0 | 0.08 |
| Lactase-like protein, isoform 1 | Q8K1F9-1 | 0 | 2 | 0 | 0.08 |
| Ras-like protein family member 10A | Q8K5A4 | 0 | 2 | 0 | 0.08 |
| Conserved oligomeric Golgi complex subunit 4 | Q8R1U1 | 0 | 2 | 0 | 0.08 |
| NEDD9-interacting protein with calponin homology and LIM domains | Q8VDP3 | 0 | 2 | 0 | 0.08 |
| Transient receptor potential cation channel subfamily M member 2 | Q91YD4 | 0 | 2 | 0 | 0.08 |
| Prenylcysteine oxidase | Q9CQF9 | 0 | 2 | 0 | 0.08 |
| Selenocysteine-specific elongation factor | Q9JHW4 | 0 | 2 | 0 | 0.08 |
| 72 kDa inositol polyphosphate 5-phosphatase | Q9JII1 | 0 | 2 | 0 | 0.08 |
| Matrix metalloproteinase-16 | Q9WTR0 | 0 | 2 | 0 | 0.08 |
