# Supplementary material for: Exosomes Released from Mycoplasma Infected Tumor Cells Activate Inhibitory B Cells
Source: PLoS One. 2012 Apr 27;7(4):e36138. doi: 10.1371/journal.pone.0036138 (PMC3338602; doi:10.1371/journal.pone.0036138)
Supplement: Table S3 — Data show the selective search results against a combined UniProt mouse protein database (03/2010) from the European Bioinformatics institute ( http://www.ebi.ac.uk/integr8 ). (DOCX) [file pone.0036138.s003.docx]

Table S3

Endogenous murine proteins down-regulated in myco+ B16 exosomes compared with myco- B16 exosomes

| **Protein** | **Accession number** | **No. of peptides identified** | | **Normalized to total peptides (%)** | |
| --- | --- | --- | --- | --- | --- |
|  |  | **Myco- Exo** | **Myco+ Exo** | **Myco- Exo** | **Myco+ Exo** |
| Transmembrane glycoprotein NMB | Q99P91 | 27 | 6 | 0.85 | 0.24 |
| 5,6-dihydroxyindole-2-carboxylic acid oxidase | P07147 | 16 | 3 | 0.51 | 0.12 |
| Multivesicular body subunit 12B | Q6KAU4 | 6 | 1 | 0.19 | 0.04 |
| Sodium/potassium-transporting ATPase subunit alpha-1 | Q8VDN2 | 30 | 5 | 0.95 | 0.20 |
| Ras-related protein Rab-7a | P51150 | 7 | 1 | 0.22 | 0.04 |
| Integrin beta-1 | P09055 | 17 | 2 | 0.54 | 0.08 |
| 4F2 cell-surface antigen heavy chain | P10852 | 13 | 0 | 0.41 | 0 |
| Melanocyte protein Pmel 17 | Q60696 | 11 | 0 | 0.35 | 0 |
| Histone H2B type 1-B | Q64475 | 11 | 0 | 0.35 | 0 |
| High affinity cationic amino acid transporter 1 | Q09143 | 10 | 0 | 0.32 | 0 |
| Histone H2A type 1-F | Q8CGP5 | 10 | 0 | 0.32 | 0 |
| Cofilin-1 OS=Mus musculus GN=Cfl1 | P18760 | 7 | 0 | 0.22 | 0 |
| Carbonic anhydrase 6 | P18761 | 7 | 0 | 0.22 | 0 |
| Solute carrier family 1 (Neutral amino acid transporter), member 5 | Q5U647 | 7 | 0 | 0.22 | 0 |
| EGF-like repeat and discoidin I-like domain-containing protein 3, isoform 1 | O35474-1 | 6 | 0 | 0.19 | 0 |
| Tubulin alpha-1B chain | P05213 | 6 | 0 | 0.19 | 0 |
| Alpha-N-acetylglucosaminidase (Sanfilippo disease IIIB) | A2BFA6 | 5 | 0 | 0.16 | 0 |
| Keratin, type II cytoskeletal 2 epidermal | Q3TTY5 | 5 | 0 | 0.16 | 0 |
| Tetraspanin-7 | Q62283 | 5 | 0 | 0.16 | 0 |
| Solute carrier family 12 member 7, isoform 1 | Q9WVL3-1 | 5 | 0 | 0.16 | 0 |
| Basigin, isoform 1 | P18572-1 | 4 | 0 | 0.13 | 0 |
| Moesin | P26041 | 4 | 0 | 0.13 | 0 |
| Synaptotagmin-4 | P40749 | 4 | 0 | 0.13 | 0 |
| M-phase inducer phosphatase 3 | P48967 | 4 | 0 | 0.13 | 0 |
| Monocarboxylate transporter 1 | P53986 | 4 | 0 | 0.13 | 0 |
| Histone H4 | P62806 | 4 | 0 | 0.13 | 0 |
| Adipocyte enhancer-binding protein 1, isoform 1 | Q640N1-1 | 4 | 0 | 0.13 | 0 |
| Centrosomal protein POC5, isoform 1 | Q9DBS8-1 | 4 | 0 | 0.13 | 0 |
| Guanine nucleotide-binding protein G(k) subunit alpha | Q9DC51 | 4 | 0 | 0.13 | 0 |
| Clk2-Scamp3 protein | B2M0S2 | 3 | 0 | 0.09 | 0 |
| Acetylcholine receptor subunit delta | P02716 | 3 | 0 | 0.09 | 0 |
| Ras-related protein Rab-11A | P62492 | 3 | 0 | 0.09 | 0 |
| Collagen alpha-2(V) chain | Q3U962 | 3 | 0 | 0.09 | 0 |
| Sorting nexin-25 | Q3ZT31 | 3 | 0 | 0.09 | 0 |
| Nuclear receptor corepressor 1, isoform 1 | Q60974-1 | 3 | 0 | 0.09 | 0 |
| Leucine-rich repeat-containing protein 16A, isoform 1 | Q6EDY6-1 | 3 | 0 | 0.09 | 0 |
| Nipped-B-like protein, isoform 1 | Q6KCD5-1 | 3 | 0 | 0.09 | 0 |
| Cysteine-rich secretory protein LCCL domain-containing 1 | Q8CGD2 | 3 | 0 | 0.09 | 0 |
| Phosphofurin acidic cluster sorting protein 1 | Q8K212 | 3 | 0 | 0.09 | 0 |
| Putative uncharacterized protein | Q8R2W4 | 3 | 0 | 0.09 | 0 |
| Vacuolar protein sorting-associated protein 28 homolog | Q9D1C8 | 3 | 0 | 0.09 | 0 |
| 6-phosphogluconate dehydrogenase, decarboxylating | Q9DCD0 | 3 | 0 | 0.09 | 0 |
| Glycoprotein-N-acetylgalactosamine 3-beta-galactosyltransferase 1 | Q9JJ06 | 3 | 0 | 0.09 | 0 |
| Zinc finger homeobox protein 4 | Q9JJN2 | 3 | 0 | 0.09 | 0 |
| Alpha-N-acetylgalactosaminide alpha-2,6-sialyltransferase 3 | Q9WUV2 | 3 | 0 | 0.09 | 0 |
| Large neutral amino acids transporter small subunit 1 | Q9Z127 | 3 | 0 | 0.09 | 0 |
